# Managing acute altitude illness

**DOI:** 10.1016/j.mmr.2026.100068

**Published:** 2026-09-29

**Authors:** Martin Burtscher, Johannes Burtscher

**Affiliations:** Institute of Sport Science, University of Innsbruck, 6020 Innsbruck, Austria; Department of Psychiatry, Psychotherapy, Psychosomatics and Medical Psychology, University Hospital for Psychiatry II, Medical University of Innsbruck, 6020 Innsbruck, Austria

**Keywords:** High altitude, Sickness, Prevention, Treatment, Acclimatization

Rapid ascent to high altitude puts non-acclimatized individuals at risk of acute altitude illness. Essential healthcare management includes individual susceptibility assessment, timely preventive measures, and appropriate treatment. Acute altitude illness can range from the usually mild acute mountain sickness (AMS) to the rare but potentially life-threatening conditions high-altitude cerebral edema (HACE) and high-altitude pulmonary edema (HAPE) [1]. AMS typically presents with headache, nausea, fatigue, and dizziness, the principal diagnostic criteria, while HACE is characterized by severe neurological symptoms, particularly ataxia, confusion, and altered consciousness, and HAPE is characterized by progressive dyspnea, reduced exercise tolerance, and cough [2]. The underlying cause of altitude illness is a reduced availability of oxygen, resulting in hypoxemia and tissue hypoxia, which trigger a complex cascade of physiological and molecular responses [1], [2]. The magnitude and interplay of these responses shape the clinical spectrum and severity of illness and define potential targets for prevention and treatment (Fig. 1).

**Fig. 1:**
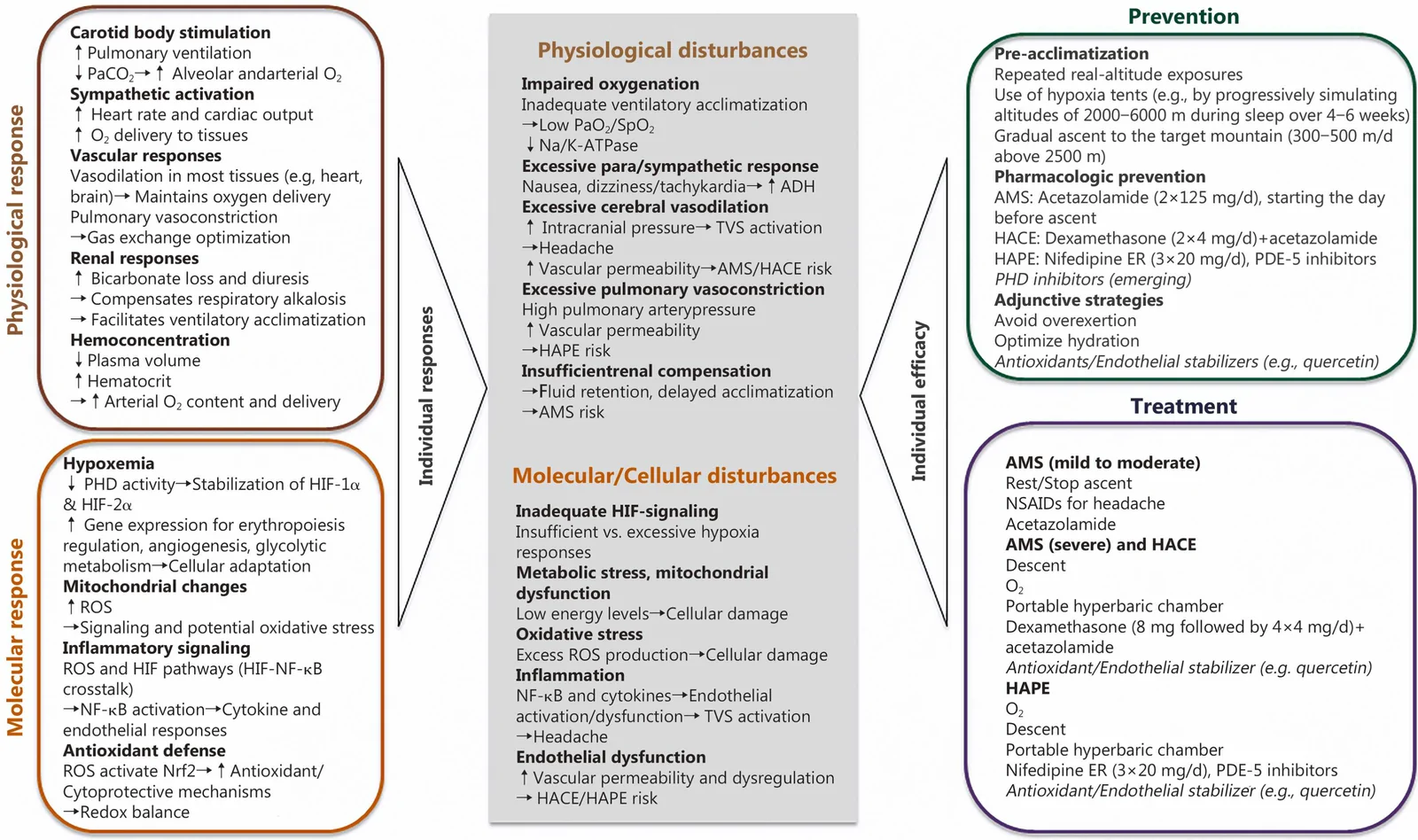
Physiological and molecular responses that compensate for reduced oxygen availability during acute exposure to high altitude, processes potentially contributing to the development of acute altitude illness, and related strategies for prevention and treatment. The arrows indicate that physiological and molecular disturbances are shaped by individual responses to hypoxia (left) and may be counteracted differently by preventive and therapeutic interventions depending on individual efficacy (right). The measures shown in italics require further validation. AMS. Acute mountain sickness; ER. Extended- release; HACE. High-altitude cerebral edema; HAPE. High-altitude pulmonary edema; HIF. Hypoxia-inducible factor; NF-κB. Nuclear factor κB; Nrf2. Nuclear factor erythroid 2-related factor 2; O_2_. Oxygen; PaCO_2_. Arterial partial pressure of carbon dioxide; PDE-5. Phosphodiesterase type 5; PHD. Prolyl hydroxylase domain protein; ROS. Reactive oxygen species; TVS. Trigeminovascular system.

Individual susceptibility, the rate and mode of ascent, and the absolute altitude attained represent major risk factors [1]. Previous experience of high altitudes may provide valuable insight into an individual’s susceptibility, while low-altitude tests remain limited in their predictive accuracy [1]. Proposed markers include a greater decline in pulse oxygen saturation (SpO₂) during exercise, particularly in men [3], and genetic variants associated with high-altitude adaptation [4].

The cornerstone of prevention is gradual ascent up the target mountain, ideally preceded by repeated altitude or hypoxia exposure (pre-acclimatization) near home, e.g., in hypoxic tents [5]. Effective hypoxia exposure protocols for individual pre-acclimatization are still being explored, but approximately 200 h of repeated hypoxia before ascent can substantially reduce the risk of altitude illness up to approximately 6000 m [5]. Pharmacologically, acetazolamide remains the only established intervention that accelerates ventilatory acclimatization [1], [2].

If AMS symptoms develop, the first measures are to stop ascending, rest, and consider symptomatic treatment with non-steroidal anti-inflammatory drugs and antiemetics [1], [2]. If AMS symptoms are severe, persist despite initial measures, or progress to HACE or HAPE, the most effective intervention is to reduce the hypoxic stimulus by descending to a lower altitude and/or administering supplemental oxygen (SpO_2_ >90%) [1], [2]. Portable hyperbaric chambers can provide a temporary alternative when descent is not immediately possible. In HAPE, pulmonary vasodilators such as nifedipine or phosphodiesterase-5 inhibitors may be considered [1], [2].

Despite the availability of several preventive and therapeutic options, their practical applicability, efficacy, and safety may be limited, particularly with regard to prolonged pre-acclimatization, descent, and pharmacological interventions. Furthermore, there are no objective biomarkers for predicting or diagnosing AMS, or the early stages of HACE or HAPE. This reflects our limited understanding of the pathophysiology of altitude illness, which may explain why there are no new treatment options despite our growing understanding of the physiological and molecular responses to hypoxia. The central targetable pathways of the response to hypoxia include the hypoxia-inducible factor/prolyl hydroxylase domain protein (HIF/PHD) oxygen-sensing system, as well as mitochondrial dysfunction, oxidative stress, inflammation, epigenetic adaptation/maladaptation, and endothelial dysfunction [6]. Modulation of HIF, especially using PHD inhibitors, is emerging as a powerful treatment option in anemia and cancers [6], and may be explored to correct inadequate HIF signaling related to altitude illness. Antioxidants such as quercetin show promise for AMS prophylaxis but require further evaluation [7]. Oxidative stress and inflammatory responses are highly intertwined [8], especially in hypoxia, and may amplify endothelial dysfunction, vascular permeability, cerebral edema, and potentially pulmonary vascular injury, key features in the development of altitude illness. Dexamethasone is an established treatment for severe AMS and HACE and attenuates inflammatory and endothelial responses, reducing cerebral edema [9]. Other potential strategies targeting oxidative stress, inflammatory signaling, mitochondrial function, or epigenetic mechanisms may offer avenues for the prevention or treatment of altitude illness, but their clinical relevance remains to be established.

An important advancement in this area is the attempt to categorize different subtypes of altitude illness based on their phenotype or pathophysiology, such as variations in cerebral trigeminovascular activation, blood-brain barrier dysfunction, edema, inadequate ventilatory acclimatization, or exaggerated inflammatory/oxidative responses. This could facilitate personalized medical approaches tailored to specific subtypes. These approaches are driven by the contemporary ability to combine large-scale omics studies [10] with in-depth physiological characterization through continuous monitoring (e.g., using wearable technology) and to analyze subtype features using state-of-the-art analytical technologies. Despite their considerable potential, these approaches have yet to translate into meaningful advances in the diagnosis and treatment of altitude illness.

Abbreviations

AMS: Acute mountain sickness

SpO₂: Pulse oxygen saturation

HACE: High-altitude cerebral edema

HAPE: High-altitude pulmonary edema

HIF: Hypoxia-inducible factor

PHD: Prolyl hydroxylase domain protein

## Declarations

### Ethics approval and consent to participate

Not applicable.

## Authors’ contributions

MB conceived the editorial and provided the first draft. MB and JB created the figure. JB provided textual input, reviewed, and revised the draft. Both authors read and approved the final manuscript.

## Funding

Not applicable.

## Competing interest

The authors declare that they have no competing interests.

## Data Availability

Not applicable.
